# Particular CSF sphingolipid patterns identify iNPH and AD patients

**DOI:** 10.1038/s41598-018-31756-0

**Published:** 2018-09-11

**Authors:** Enrica Torretta, Beatrice Arosio, Pietro Barbacini, Martina Casati, Daniele Capitanio, Roberta Mancuso, Daniela Mari, Matteo Cesari, Mario Clerici, Cecilia Gelfi

**Affiliations:** 1 Department of Biomedical Sciences for Health, University of Milan, Segrate (Milan), Italy; 2 Geriatric Unit, Department of Medical Sciences and Community Health, University of Milan, Milan, Italy; 3 Fondazione IRCCS Ca’ Granda – Ospedale Maggiore Policlinico, Milan, Italy; 4 Don C Gnocchi Foundation IRCCS, Milan, Italy; 5 Department of Physiopathology and Transplants, University of Milan, Milan, Italy; 6 Clinical Proteomics Unit, Scientific Institute for Research, Hospitalization and Health Care (IRCCS) Policlinico San Donato, San Donato Milanese (Milan), Italy

**Keywords:** Alzheimer's disease, Hydrocephalus

## Abstract

Idiopathic normal pressure hydrocephalus (iNPH) is characterized by reversible neurological symptoms due to an impairment in cerebrospinal fluid (CSF) clearance. In these patients, cognitive functions are severely impaired, with a scenario similar to Alzheimer’s disease (AD), making the differential diagnosis difficult and highlighting the need of new markers. We analyzed the composition of sphingolipids (SLs) in serum, by combining a single phase extraction with a high-performance thin-layer chromatography (HPTLC) primuline-profiling, and, in CSF, by MALDI profiling and LC-MS. Ceramides and sphingomyelins (SMs) were similar in serum of iNPH and AD patients compared to healthy controls, whereas, in CSF, MALDI profiling indicated that: 1) SM C24:1 is significantly decreased in AD compared to iNPH patients and controls (Kruskal-Wallis p-value < 0.00001); 2) phosphatidylcholine (PC) 36:2 is increased in iNPH patients (p-value < 0.001). LC-MS identified an increasing trend of Cer C24:0 and of a set of SMs in patients with AD, a significant decrease of sphingosine-1-phosphate (S1P) (t-test p-value 0.0325) and an increase of glucosylceramide (GlcCer) C24:0 (p-value 0.0037) in AD compared to iNPH patients. In conclusion CSF PC 36:2, SM C24:1, S1P, and GlcCer can contribute to improve the differential diagnosis of patients with iNPH or AD and foster preventive therapeutic strategies in the early phase of the disease.

## Introduction

Alzheimer’s Disease (AD) is a neurodegenerative disorder characterized at the onset by memory loss, followed by spatial disorientation and wandering, difficulty in communication, mood changes, and behavioral disorders at advanced phases. The prevalence of AD is continuously increasing, due to earlier diagnosis and increased life expectancy. It is estimated a number of 13.2 million patients by the year 2050^1^, obviously representing a major challenge for the sustainability of healthcare systems.

The lack of knowledge about the pathophysiological mechanisms of the disease and the absence of biomarkers able to predict the evolution of the disease from the prodromal phases to the overt manifested AD, substantially affect preventive and therapeutic strategies. Current FDA-approved drugs (e.g. cholinesterase inhibitors, NMDA-receptor agonists) are only symptomatic and not disease-modifiers.

AD diagnosis is currently based on combination of information coming from the patient’s disease history, the assessment of the cognitive function, and neuroimaging (CT, MRI and PET)^2^. The measurement of levels of amyloid-β (Aβ42), total-tau (t-tau), and phospho-tau181 (p-tau) in cerebrospinal fluid (CSF) can support the differential diagnosis of AD, and support the prediction of those individuals with mild cognitive impairment (MCI) potentially converting to AD in the future^3–5^.

Idiopathic normal pressure hydrocephalus (iNPH) is a disease characterized by reversible neurological symptoms due to impairment of CSF clearance^6,7^. Patients show a severely cognitive impaired function, often difficult to differentiate from AD^8^. Initially, the disease was described as a triad of symptoms: cognitive impairment, urine incontinence and gait disturbance^9^. iNPH symptoms can be alleviated by shunt surgery^10^. Notably, in 18% to 42% of cases, AD represents a comorbidity of iNPH^11,12^. It is noteworthy that AD and iNPH patients present similar clinical features and molecular characteristics^13^, including amyloid deposition^14,15^ and t-tau and p-tau dysregulation. However, quantitative results of t-tau and p-tau on iNPH patients are contradictory, due to high interindividual variability indicating the need of other markers for improving the differential diagnosis^16–20^ and to predict the evolution toward cognitive impairment (CI). Starting treatments in the early phase will counteract the stabilization of CI in iNPH or contribute to slow it down in AD.

In a previous study^21^, we described the differential protein profile and the APOA1 proteoform composition in CSF of iNPH and AD patients; results showed that the CSF profiling of low abundant low molecular weight proteins of iNPH was similar to that of healthy controls, indicating that alterations in CSF proteins are typical of AD patients.

To shed light on the pathogenesis of AD and iNPH we analyzed the lipidome and, specifically, the composition of sphingolipids (SLs) in these patients. SLs are structural components of the plasma membrane involved in the regulation of membrane fluidity, intercellular communication, signal transduction and cell activation. Alterations in the metabolism of these lipids is known to be correlated with neurodegenerative disease, including AD, and changes in SLs level were recently described in MCI and AD patients^22,23^. However, comparative studies exploring levels of circulatory SLs in a transient state of cognitive impairment such in patients with iNPH are lacking. Herein, we investigated SLs flux in iNPH and AD patients by combining a single phase extraction method with a HPTLC primuline-profiling in serum and by MALDI profiling and LC-MS in CSF.

## Results

Serum and CSF samples were collected from 10 patients affected by iNPH (M/F-5/5, age 85 ± 9.6), and 16 patients with AD disease (M/F-7/9, age 76 ± 3.8). CSF samples from 10 control subjects with no history of neurogical disorders (M/F- 6/4, age 75 ± 4.7) were also analyzed. Concentration of free Aβ42, t-tau and p-tau were measured in CSF. Free Aβ42 values were pathological for patients with AD (392 pg/mL) and normal for healthy subjects (1082 pg/mL) and patients affected by iNPH (790 pg/mL). The same was observed for t-Tau (774 pg/mL in patients with AD, 103 pg/mL in healthy subjects and 157 pg/mL in patients with iNPH) and for p-Tau (84 pg/mL for patients with AD and 26 pg/mL for healthy subjects and patients with iNPH). Patients’ description is shown in Table 1 and in Table S1.

**Table 1:** Participants’ characteristics.

	Healthy subjects, N = 10 (M = 6/F = 4)	iNPH patients, N = 10 (M = 5, F = 5)	AD patients, N = 16 (M = 7, F = 9)
Age, years (median, min-max, standard deviation)	75 (68–84) ± 4.7	85 (70–100) ± 9.6	76 (70–82) ± 3.8
Aβ, pg/mL (median, min-max, s.d.)	1082 (453–1515) ± 467	790 (718–1248) ± 267	392 (226–563) ± 96
Tau, pg/mL (median, min-max, s.d.)	103 (49–507) ± 160	157 (75–284) ± 73.4	774 (208–1420) ± 394
p-Tau, pg/mL (median, min-max, s.d.)	26 (7–80) ± 24	26 (15–42) ± 9.2	84 (28–136) ± 31.7
MMSE	//	28 (17–29) ± 3.9	22 (13–27) ± 4.3

### Sphingolipid profiling in serum

Differences in the sphingolipid profiles in serum of control subjects and of iNPH and AD patients were investigated by HPTLC-densitometry and FDIC (fluorescence detection by intensity changes) emission after primuline staining.

After extraction, sphingolipids from 10 controls, 10 iNPH and 16 AD sera were separated on HPTLC plates and SL bands were compared by densitometry.

From primuline stained HPTLC plates, bands corresponding to Cers and SMs were identified by Rf comparison with standards, carrying a variation coefficient (CV) of 6–7%; bands with Rf = 0.923, Rf = 0.858, Rf = 0.176 and Rf = 0.152 were respectively attributed to long chain Cers, short chain Cers, long chain SMs and short chain SMs.

**Figure 1:**
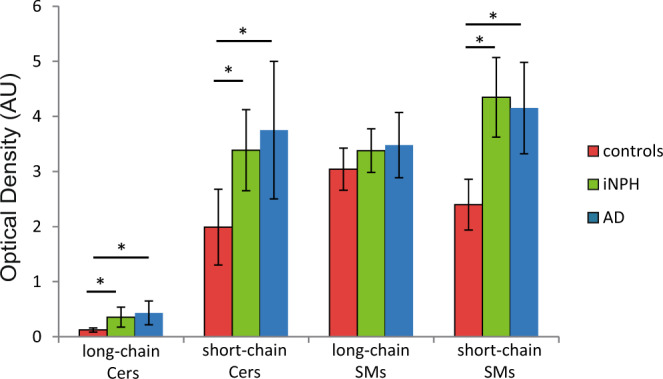
Comparison of ceramides and SMs circulating levels in sera from controls (n = 10), iNPH (n = 10) and AD (n = 16) patients by primuline/HPTLC densitometry. Long and short chain Cers were statistically increased (Kruskal-Wallis p-value < 0.001) in sera from iNPH (Dunn’s test p-value < 0.05) and AD (Dunn’s test p-value < 0.05) compared to control subjects. Long chain SMs were unchanged whereas short chain SMs were statistically higher (Kruskal-Wallis p-value < 0.001) in sera from iNPH (Dunn’s test p-value < 0.05) and AD (Dunn’s test p-value < 0.05) compared to controls.

Primuline quantitative staining revealed a statistically significant increase of long and short chains Cers (Kruskal-Wallis p-value < 0.001) in patients with iNPH and with AD compared to controls. Long chain SMs were unchanged whereas short chain SMs increased both in iNPH and AD patients (Kruskal-Wallis p-value < 0.001) compared to controls. No significant changes were detected between iNPH and AD patients in blood (Fig. 1).

### CSF MALDI profiling analysis

**Figure 2:**
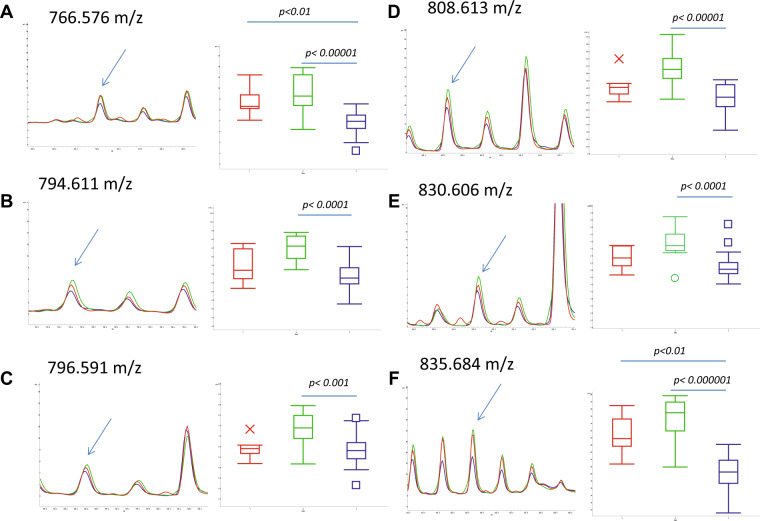
Close-up of ClinProTools average spectra for control subjects (red), iNPH (green) and AD patients (blue), showing best separating peaks. Box-plots are shown for each peak. CSF samples were extracted with chloroform/methanol and lipid fractions were spotted onto the AnchorChip target in four replicates; spectra have been acquired in reflectron positive mode in the m/z range 200–2000. No changed peaks was detected in the pairwise comparison of control subjects versus iNPH patients. Peaks at 766.57 m/z (**A**) and at 835.68 m/z (**F**) were statistically changed between control subjects and AD patients and between iNPH and AD patients. Conversely, peaks at 794.61 m/z (**B**), 796.59 m/z (**C**), 808.61 m/z (**D**) and 830.61 m/z (**E**) statistically differ between iNPH and AD patients, only.

To get better insights into the differences of the sphingolipid profiles related to the neurodegenerative process compared to controls, lipids were extracted from CSF of AD and iNPH patients and from age-matched control subjects. The low abundance of SLs in CSF hampered their detection by primuline staining, so CSF extracted organic phases were loaded with DHB matrix on an Anchorchip MALDI target for spectra acquisition and MALDI profiling analysis. Statistical analysis performed by ClinProTools software detected 6 “best separating peaks” (Kruskal-Wallis p-value < 0.01, with CV < 20%) (Table 2). To determine which peak differs in which groups, a pairwise comparison was performed. A recent study on the same group of patients demonstrate that the protein/peptide CSF profiles of healthy controls and of patients with iNPH didn’t show any statistically significant difference^21^. Same results were obtained when MALDI profiling of the lipid organic phases were achieved from controls and iNPH patients, further supporting the use of these samples in comparative studies of AD patients. Conversely, comparing organic phases from AD, iNPH patients and controls, several differences were observed, particularly peaks at 766.58 m/z and at 835.68 m/z were statistically decreased in AD patients compared to control subjects (with AUC values of 0.910156 and 0.953125, respectively), and to iNPH patients (Fig. 2A,F). Interestingly, peaks at 794.61 m/z, 796.59 m/z, 808.61 m/z and 830.61 m/z were statistically changed between iNPH and AD patients, only (Fig. 2B–E). AUC values of best separating peaks for patients with iNPH vs AD comparison are shown in Table 3. All peaks were underexpressed in patients with AD.

**Table 2:** List of ‘best separating’ peaks (Kruskal-Wallis p-value < 0.01, CV < 20%) obtained through sphingolipid MALDI profiling analysis on CSF samples taken from control subjects and iNPH and AD patients.

Peak Mass	Kruskal-Wallis P-Value
766.576	0.000059
794.611	0.000212
796.591	0.00261
808.613	0.000102
830.606	0.000372
835.684	0.00000355

**Table 3:** AUC values of best separating peaks in the pairwise comparison of iNPH vs AD.

Peak Mass	AUC Value (iNPH vs AD)
766.576	0.929688
794.611	0.900391
796.591	0.849609
808.613	0.935547
830.606	0.902344
835.684	0.974609

### Best separating peaks identification

The identification of best separating peaks was performed by MALDI-MS/MS. Based on the m/z ratio of parent ion and on fragment ions assignment, the peak at 835.68 m/z was identified as a sphingomyelin (SM d18:1 C24:1 [M + Na]^+^) (Fig. 1S). To validate this identification, the organic phase was submitted to alkaline hydrolysis, which removes all phospholipids except sphingomyelin. The latter is characterized by an amide group resistant to the hydroxide nucleophilic attack. Spectra of hydrolized sample achieved by MALDI identified a signal at 835.68 m/z confirming sphingomyelin identification (Fig. 2S).

**Figure 3:**
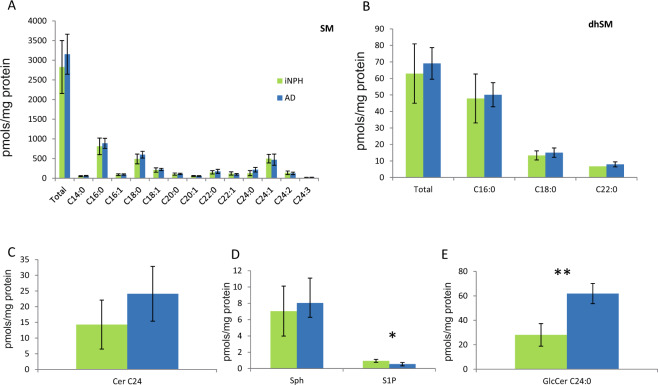
LC-MS analysis sphingolipid levels in CSF from iNPH and AD patients. Total SMs (**A**) and dhSMs (**B**), as well as Cer C24:0 (**C**), tend to increase in AD patients compared to iNPH patients. S1P (**D**) decreased in AD compared to iNPH patients (t-test p-value < 0.05). GlcCer C24:0 (**E**) statistically increased in AD compared to iNPH patients (t-test p-value < 0.01).

**Figure 4:**
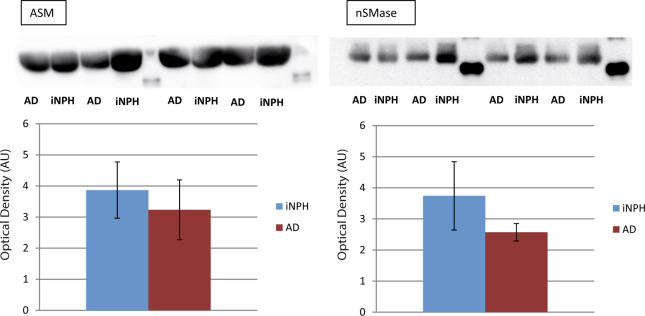
Immunoblot closeups (cropped images; full lenght blot is included as Supplementary Information) and histograms of protein expression levels are shown for neutral (nSMase) and acid Sphingomyelinase (ASM) in CSF from iNPH and AD patients. Both neutral and acid Sphingomyelinase tended to decrease in AD patients compared to iNPH controls. Ceramidase was not appreciably detected. Data were normalized against the total amount of loaded proteins stained with Sypro Ruby and reported as mean ± SD.

Other differentially changed peaks were identified as phosphatidylcholines (PCs) (Table 4). The peak at 808.61 m/z was attributed to PC 36:2 [M + Na]^+^ through the NIST MS Search v 2.2 software (Fig. 3S), whereas for peaks at 766.58 m/z and at 796.59 m/z, the software provided multiple structures, as the m/z peak ratio overlaps with different phosphatidylcholines (Fig. 4S).

**Table 4:** Proposed structures for best separating peaks at 835.684, 808.613, 766.576, 769.591 m/z.

m/z	theorical m/z (*)	Identification/Proposed structures
835.684	835.666	SM d18:1 C24:1 [M + Na]^+^
808.613	808.583	PC 36:2 [M + Na]^+^
766.576	766.551	PC(O-16:0) [M + H]^+^
		PC 35:5 [M + H]^+^
		PC 33:2 [M+Na]^+^
796.591	796.554	PC 36:8 [M+Na]^+^
		PC 37:4 [M+H]^+^
		PC 35:1 [M+Na]^+^

### LC-MS analysis

It is well known that MALDI profiling cannot provide the detection of all SL species due to the ion suppression caused by phospholipids or by peaks generated by the DHB matrix. To get better insights into the sphingolipidome of CSF in patients with iNPH and AD, 3 sub-pools per group were created and analysed by LC-MS. The sub-pooling has been largely adopted in proteomic studies to reduce the variance among biological groups increasing the power to detect more significant changes when few samples are available and the variance is high. In the present study we adopted the same approach to enlarge the set of differentially expressed SL species due to the wide inter individual variability of serum sphingolipidome taking into account results from the well standardized primuline staining shown in Fig. 1 and considering the absence of any reference data.

Quantitation of SMs, dhSMs and Cer (Fig. 3A–C) confirmed the increasing trend observed for SMs (except for SM C24:1) and dhSM, although not statistically significant, together with an increasing trend of Cer C24:0 in AD compared to iNPH patients. Sphingosine-1-phosphate (S1P) (Fig. 3D) was statistically decreased in CSF of AD compared to iNPH patients (t-test p-value 0.0325), whereas glucosylceramide C24:0 (Fig. 3E) was statistically increased in AD compared to iNPH patients (t-test p-value 0.0037).

### Western blot analysis

Data from LC-MS and MALDI profiling analysis showed differences between patients with AD and iNPH, and provided a picture of the complexity of metabolic pathways that led to sphingolipidomics differences. To determine and validate, at least indirectly, changes observed in the sphingolipid profiles of our patients, the sphingomyelinase and ceramidase, involved in the metabolic pathway of SM and Cer, levels were assessed by immunoblotting in CSF of AD and iNPH patients. Quantitation of nSMase (neutral sphingomyelinase) and ASM (acid sphingomyelinase) (Fig. 4) revealed a decreasing trend, although not statistically significant, in AD patients compared to iNPH, whereas, Anti-Ceramidase antibody (anti-ASAH2) was tested on the same sub-pools but ceramidase was, in our hands, undetectable in CSF samples.

**Figure 5:**
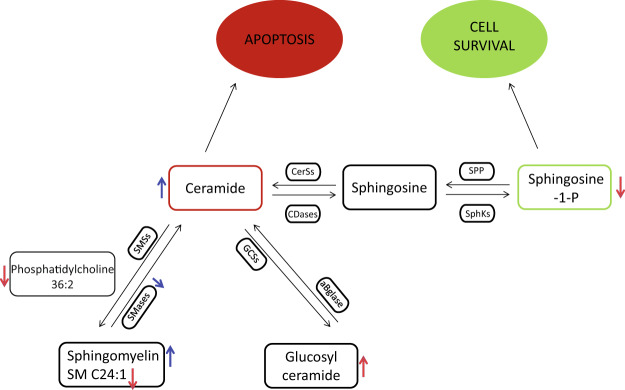
Schematic results summary. Total SMs tend to increase in patients with AD compared to patients with iNPH, whereas SM C24:1 is significantly decreased; both the SMases (nSMase and ASM) result to be decreased, whereas Cer C24:0 tend to increase. Sphingosine-1-phosphate(S1P) is significantly reduced in AD compared to iNPH patients whereas glucosylceramide (GlcCer)C24:0 is augmented. Red arrows indicate a statistical significant result, whereas blue arrows indicate an increasing/decreasing trend.

Figure 5 provides an overview of results characterizing the CSF SLs profiling of iNPH and AD patients. PC 36:2 and SM C24:1 were significantly decreased in AD compared to iNPH patients whereas total SMs tended to increase; nSMase and ASM revealed a decreasing trend, whereas Cer C24:0 tended to augment. Sphingosine-1-phosphate (S1P) was significantly reduced in AD compared to iNPH patients whereas glucosylceramide (GlcCer) C24:0 increased.

## Discussion

In the present study, variations of sphingolipid levels in CSF and serum were for the first time investigated in patients with iNPH. Our previous study demonstrated that patients with iNPH shares a CSF MALDI low molecular weight protein profile similar to healthy controls and these patients can be considered a good reference for comparative study due to well matched physiological characteristics and the availability of CSF, routinely collected for diagnosis and therapy compared to healthy subjects.

In the present study, the “sphingolipidome” analysis was performed combining different technologies to enlarge the data set of differentially expressed species. It is known that the use of different techniques adopting different separative steps (i.e. HPTLC vs HPLC) prior MS analysis and mass spectrometers (MALDI vs. LC-MS), allows to enlarge the data set of recognized species. In addition, due to the absence of an independent assay for data validation, being the use of antigen antibody detection hampered by mimicry exerted by antibodies^24,25^, the adoption of multiple technological approaches further supports the obtained semi quantitative data.

In serum, primuline staining after HPTLC indicated that both patients with iNPH and AD share the same increase of long chain and short chain Cers together with short chain SMs compared to controls. Blood sphingolipids may directly reflect brain and CSF SLs levels, due to the SLs crossing of the blood brain and of the blood-spinal cord barriers, or peripheral SLs may be associated with atherosclerosis, insulin resistance and diabetes, all of which are known risk factors for AD^26^.

Results indicate that the pool of Cers and of long chain SMs species, belonging from different compartment and released in blood, are dysregulated both in patients with iNPH and AD, however, to decipher the contribution to neurodegeneration of specific chains and to highlight which, among other molecules, characterizes patients with iNPH and AD, a refined analysis of CSF was used.

The MALDI CSF profiling indicated a decrease of peaks at 766.58 m/z (Fig. 2A) and at 835.68 m/z, identified as SM C24:1, in AD patients compared to iNPH patients and controls, whereas peaks at 794.61 m/z, 796.59 m/z, 808.61 m/z (identified as PC 36:2 [M + Na]^+^) and 830.61 m/z were statistically increased in patients with iNPH compared to AD (Fig. 1B–E). LC-MS analysis identified a set of SMs, not identified by MALDI, increased in patient with AD, together with a trend of increased levels of Cer C24:0. Importantly, in CSF, S1P levels were found statistically decreased in AD compared to iNPH affected patients, conversely GlcCer C24:0 was statistically increased in patients with AD.

Fonteh *et al*.^27^ described in CSF a decrease of total SM in CSF of AD patients compared to MCI and control subjects whereas Kosicek *et al*.^28^ identified in cognitive normal vs prodromal AD a significant increase of SMs in patients with AD (C14, C16, C20, C22 and C24 chains), and a non statistically significant decline of SMs in patients with mild to moderate AD compared to cognitively normal controls. However, the mechanism for SM increase in the prodromal phase of the disease is still unclear. Authors hypothesize that this early elevation of SMs in CSF of AD patients could result from an increased level of Cers utilized for SMs synthesis. Similarly, Mielke *et al*. found a correlation between sphingomyelin and Aβ values, total Tau and some forms of p-Tau in CSF of healthy individuals with parental history of AD^29^. In brain tissue, increased Cer levels and reduced SM concentrations were described in human gray matter of frontotemporal area in AD patients^30–32^. The increased levels of Cers, both in CSF and brain tissue, suggest a translocation of a-SMase to the plasma membrane leading to Cers over-production and SMs reduction^33^. Unfortunately, from our results, any apparent change has been reported for n-SMase^33^ indicating that the mechanism is more complex. It should also be considered that the concentration of most lipids in the human brain decrease after the age of 50. Phosphatidylinositol (PI), phosphatidylethanolamine (PE) and phosphatidylcholine (PC) brain levels decrease very slowly with age, with less than 10% loss in the range of 40 to 100 years old^34^. A decrease of PC^35,36^ has been also reported in patients with AD. Interestingly, in the early stage of AD, brain levels of other phospholipid classes (PC, PI and PE) in both white and gray matter appeared to be unchanged^37^. Unchanged PC levels, but decreased lysoPC/PC ratio and elevation of different PC metabolites have been reported in the CSF of AD patients compared to memory complaints non demented patients^38^ suggesting an increased hydrolysis of PC in patients with AD^39^. Noteworthy, a number of studies described phospholipid alterations in brains of AD patients, but only few studies described changes of this class of molecules in CSF and data provided by the present study on iNPH patients, are new. S1P is a protective molecule, favoring cell renewal and survival, produced by the action of ceramidase that enhances ceramide catabolism and formation of its anti-apoptotic metabolite^40^. The latter is decreased in CSF from AD affected patients compared to iNPH patients indicating that the negative effects of ceramide accumulation cannot be prevented by S1P. To our knowledge, this is the first time that S1P decrement has been detected in CSF of AD patients^41,42^ since a decrement of S1P was described in the hippocampus and temporal cortex of AD human brain tissues, only^42^, providing further support to this molecule as putative biomarker of cognitive impairment. To establish the relationship between S1P decrement and AD, verification studies are currently ongoing. S1P-based signaling through S1P receptor 1 (S1PR1) rapidly and reversely reduces basal P-glycoprotein transport activity at the blood-brain and blood-spinal cord barriers and the S1P efflux from brain and spinal cord endothelial cells is mediated by the multidrug resistance-associated protein 1 (Mrp1, Abcc1)^43^. Furthermore, the activation of the proinflammatory pathway (TNF-α), typical of AD, decreases the P-glycoprotein activity and it has been described that iNPH patients show higher levels of CSF TNF-α compared to MCI subjects^44^ leading to speculate on the role of SP1 as a possible marker for differential diagnosis but also in this case results are contradictory since other publications describe low^45^ or unchanged^46^ levels of TNF-α, suggesting that also in this case the inter individual variability plays a major role.

Another novelty introduced by this study is the increment of GlcCer C24:0 detected in the CSF of AD patients. This result can be directly associated to increased levels of Cer, a precursor of GlcCer. The GlcCer increment was not observed in patients with iNPH despite increased levels of Cers. It can be hypothesized that after the translocation of a-SMase to the plasma membrane, that leads to Cer over-production and SM reduction, the higher levels of Cer stimulates the activity of ceramide glucosyltrasferase, accounting for increased GlcCer in AD.

Based on the present results, we wondered which was the contribution of GlcCer in this context. This molecules has been recently described as associated to alpha synuclein misfolded tetramer formation in Parkinson’s disease (PD)^47^ suggesting that GLSs accumulation is sufficient to increase the susceptibility of neurons to cytotoxicity. Another recent paper by Friederike Zunke *et al*.^48^ highlighted the role of GlcCer accumulation in synuclein conformers formation, however the increase of GlcCer and the GlcCer/GalCer ratio assessed in 26 PD patients with different disease stages by LC-MS/MS, indicated a trend not statistically significant, suggesting that the interindividual variability of these molecules is high and a number of other molecules should be associated to link their variation to disease progression^49^. In this context, the level of ApoA1proteoforms, described in our previous study as significantly decreased in AD compared to iNPH and controls could be good candidates to be assessed in association with increased levels of GlcCer in patients with AD. Further studies are ongoing to verify the association of ApoA1 and SLs dysregulation in MCI patients. The GlcCer accumulation, together with S1P decrement in AD can be associated to apoptosis of neuronal cells, not present in iNPH in which the mechanism of autophagy is probably maintained, studies are in progress on this direction. To contribute to shed light in the intricate picture of sphingolipids, the antigen-antibody detection of enzymes involved in Cer and SM metabolism indicated a decreased trend of acidic and neutral SMase supporting that increased levels of several species of SM, detected by LC-MS, could be associated to the decreased trend of these enzymes. Unfortunately for ceramidase, a faint band, not quantitable was detected in patients with iNPH only, whereas this band was absent in AD patients (data not shown). To detect this enzyme a larger sample loading not affordable with our CSF sample volume is needed, leaving this question still open. Beside the sample volume availability, another limitation of the present study is the number of available samples for verification studies in which a larger number of patients and controls are needed to precisely define the role of SLs and ApoA1 in AD as putative markers for AD diagnosis. Verification studies are in progress to confirm the increment of Cer, GlcCer and of same species of SM in patient with AD compared to patients with iNPH by MRM based MS analysis of CSF, in addition proteopetides of SMase and ceramidases will be quantitated adopting the same technological approach with the aim to build a multiple test able to contribute to better diagnosis of AD and iNPH syndromes.

In conclusion, as summarized in Fig. 5, the present results suggest that levels of Cers, S1P, SMs and GlcCer in CSF can contribute to the differential analysis of patients with iNPH vs. AD and can potentially be predictive to a co-morbidity of AD in iNPH patients.

## Materials and Methods

### Study participants and sample collection

Serum and CSF samples from age and sex-matched control subjects, patients with idiopathic normal pressure hydrocephalus (iNPH) disease, and patients with Alzheimer’s (AD) disease, were collected at the Geriatric Unit of the Policlinico Hospital in Milan, Italy. The present study conforms to the principles of Helsinki Declaration, and the study protocol received approval from the Ethical Committee of Fondazione Ca’ Granda IRCCS Ospedale Maggiore Policlinico, Milan, Italy. Informed consent was obtained from either patients or legal representatives. Data about their medical history, physical and neurological examination, neurocognitive evaluation (Mini-Mental State Examination), computed tomography or MRI scan, and screening laboratory tests consisting in the assessment of tau, phospho-tau (p-tau), and amyloid-β (Aβ) proteins levels by ELISA (Innogenetics) were collected. AD patients fulfilled the Dubois *et al*. criteria^50^. The iNPH subjects were diagnosed according to International Guidelines by Relkin N^10^. Control subjects were likewise examined to exclude the presence of neurological and cognitive disorders. The samples were aliquoted into 0.5 mL polypropylene storage tubes, and stored in a −80 °C freezer until analysis. Protein assays were performed using a bicinchoninic acid protein assay kit (Pierce Biotechnology, Inc., Rockford, IL, USA) with bovine serum albumin as a standard.

### Reagents/Chemical

Methanol, ethanol, HPLC-analytical grade chloroform (CHCl_3_), LC-MS grade water and LC-MS grade acetonitrile (ACN), acetone, 2,5-dihydroxybenzoic acid (DHB) matrix, 3,5-Di-tert-4-butylhydroxytoluene (BHT) and primuline dye were from Sigma-Aldrich (Saint Louis, MO, USA). 1,2-Dipalmitoyl-sn-Glycero-3-Phosphocholine (DPPC), Cardiolipin (Heart, Bovine), 1,2-Dipalmitoyl-sn-Glycero-3-Phosphoethanolamine (DPPE), Ceramide d18:1 C16:0 and Cer d18:1 C24:0 standards were from Avanti Polar Lipids (Alabaster, Alabama, USA). Sphingolipid mixture (containing Sphingomyelin, Sulfatides and Cerebrosides-HexCers-), Neutral Glycosphingolipid Mixture (including Globoside -Gb4Cer-, Ceramide trihexoside -Gb3Cer-, LactosylCeramide -LacCer- and HexCers), Monosialoganglioside mixture (containing GM1, GM2 and GM3) and Disialoganglioside mixture (containing GD1a, GD1b and GD3) were from Matreya LLC (Pleasant Gap, PA, USA).

### Lipid extraction

Lipids were extracted from serum according to the procedure of Zahir H. Alshehry *et al*.^51^. Serum aliquots of 100 µL were mixed with 1 ml of methanol/buthanol 1:1 (v/v) containing ammonium formate 5 mM. Mixtures were vortexed for 10 seconds, sonicated for 1 hour in a sonic water bath (20 °C) and centrifuged for 10 minutes at 16000 g (20 °C). Liquid phases were collected and transferred in borosilicate glass vials with Teflon-lined caps (VWR, Radnor, PA) and, after nitrogen-flux drying, re-suspended in 150 μL of chloroform/methanol 2:1 (v/v) containing BHT 0.01% (w/v) and stored at −20 °C. Total protein content was determined, according to manufacturer’s instructions, for each sample prior to lipid extraction, using Pierce™ BCA Protein Assay Kit (Pierce Biotechnology, Inc., Rockford, IL, USA).

CSF lipids were extracted using a modified Bligh and Dyer procedure^29^. Briefly, 100 µl of CSF were mixed with 300 µl of methanol containing ammonium formate (53 mM) and BHT 0.01% weight/volume (w/v). After mixture vortexing, 400 µL of chloroform were added. The mixture was centrifuged at 2000 g for 15 minutes. The chloroform layer was carefully removed and dried in a nitrogen evaporator. The lipid content was normalized in relation to total protein content and dried extracts were resuspended in a volume of 1 µL of chloroform/methanol (2:1, v/v) per µg protein and stored in borosilicate glass vials with Teflon-lined caps (VWR, Radnor, PA).

### HPTLC-Primuline

Aluminium backed HPTLC silica plates 200 × 100 mm (MERK, Billerica, Massachusetts), were initially developed in chloroform/methanol 1:1 (v/v), and dried at 120 °C for 20 minutes. Each sample (100 µg of total protein) was loaded in duplicate on HPTLC plates using Linomat 5 semiautomatic TLC spotter (CAMAG, Switzerland) (lane width: 6 mm, dosage speed: 150nL/s). Two different standard mixtures were loaded on HPTLC plates lateral edges. First mixture consisted of SM, GlcCer, Cer C16 and C24 standards; the second one was made of DPPE and DPPC. After loading, HPTLC plates were developed in chloroform/methanol/water 55:20:3 (v/v/v), using Automatic Developing Chamber 2 (CAMAG, Switzerland) with settings as follows: plate size 20 × 10 cm, pre-drying, control humidity 3 minutes, saturation time 20 minutes, plate preconditioning time 1 minute, migration distance 65 mm and drying time 1 minute. Developed plates were sprayed with a solution of primuline yellow dye, 5 mg/100 ml in propan-2-one/water 80:20 (v/v) and dried under a fume hood. Images from primuline-stained plates were acquired using Ettan DIGE Imager (GE Healthcare, Chicago, IL, USA). Imager settings were: pixel size 100 µm, excitation filter 480/30, emission filter 592/25. Acquired images were analyzed using ImageQuant software v.8.1 (GE Healthcare, Chicago, IL, USA) setting as follows: manual lane selection, rolling ball baseline subtraction and automatic band detection. Data were exported in Excel format. Excel data were analyzed using the software SigmaStat v.3.5 build.3.5.0.74 (Systat Software Inc., California, USA).

### CSF MALDI Profiling

0.5 µL of 2,5 dihydroxybenzoic acid (DHB) matrix at 20 mg/ml in 70% acetonitrile were loaded on Anchorchip target (600–384 target, Bruker Daltonics) and 0.5 µl of organic phases obtained from lipid extraction and drying of CSF samples, were spotted in four replicates on the matrix layer. A calibration solution was prepared by mixing PC, ceramides and cardiolipin standards, and loaded on calibration spots, with the same procedure followed for samples. The mixture was let to dry at room temperature. Spectra were acquired in reflectron positive modality using an Ultraflex III mass spectrometer equipped with Smartbeam laser (frequency of 100 Hz, Bruker Daltonics), Flex Control software v. 3.3, and Flex Analysis software v.3.3 (Bruker Daltonics). The spectrometer settings were: ion source 1, 25 kV; ion source 2, 21.67 kV; lens, 9.3 kV; reflector 26.3 kV; reflector 2 13.7 kV; mass suppression up to m/z 200; detector gain voltage, 1645 V; electronic gain, 100 mV/full scale. Spectra were collected using an automatic software, AutoXecute (Bruker Daltonics), whose parameters were the following: fuzzy control, off; laser power, 60%; total laser shots, 1000; random walk movement (20 shots per raster spot). CSF profiling mass spectra were analyzed by ClinProTools software v.2.2 (Bruker Daltonics) using the following spectra preparation parameters: 18000 resolution, Top Hat Baseline, 7% minimal baseline width, Savitsky–Golay smoothing.

ClinProTools’ statistics was performed by Wilcoxon’s t-test (2 classes) or Kruskal-Wallis test (>2 classes) through which a list of best separating peaks (p-value < 0.01) was generated.

### Best separating peaks identification

Ions of interest were subjected to MALDI-TOF/TOF for structure determination and eventual identification. Mass signal of parent ions were isolated and fragmented in LIFT operation mode. The nomenclature introduced by Domon and Costello^52^ and Adams and Ann^53^ was used for the assignment of the fragments ions. NIST MS Search v 2.2 software was used as a support for peaks identification: MS/MS spectra were loaded in the software and compared with those included in NIST/EPA/NIH Mass Spectral Library (NIST 14) and in LipidBlast Library^54^.

To confirm the identification of sphingomyelin, the organic phase was submitted to an alkaline treatment to remove glycerophospholipids. Briefly, it was resuspended in 100 µL of CHCl_3_ and 100 µL of 0.6 N NaOH in methanol and allowed to stand at 37 °C for 2 hours. The reaction was blocked by adding 120 µL of 0.5 M HCl in methanol. The sample was then submitted to another phase separation and the new organic phase was loaded on an Anchorchip target for spectra acquisition.

### LC-MS analysis

For LC-MS analysis, sera were randomly sub-pooled into 3 groups of iNPH and 3 of AD CSF samples. The sub-pooling was adopted as a method to reduce the variance among biological groups increasing the power to detect changes when few samples are available and the variance is high^55,56^. Sphingolipid extracts were analysed in presence of N-lauroyl-D-erythro-sphingosine, N-lauroyl-D-erythro-sphinganine, N-lauroyl-D-erythro-sphingosylphosphorylcholine N-lauroyl-D-erythro-sphinganylphosphorylcholine, D-glucosyl-ß−1,1′-N-lauroyl-D-erythro-sphingosine, D-lactosyl-ß-1,1′ N-lauroyl-D-erythro-sphingosine and C17 d-erythro-dihydrosphingosine-1-phosphate (0.5 nmol each, as internal standards, prepared as described by Merrill *et al*.^57^ and analysed by Waters Aquity UPLC system connected to a Waters LCT Premier orthogonal accelerated time of flight mass spectrometer (Waters, Millford, MA), operating in positive electrospray ionisation mode. Full scan spectra were acquired from 50 to 1500 Da, and individual spectra were summed to produce data points each 0.2 s. Mass accuracy and reproducibility were maintained by using an independent reference spray by the LockSpray interference. C8 Acquity UPLC BEH (Waters), 100 mm length, 2.1 mm outer diameter, i.d., 1.7 mm, analytical column was employed. Mobile phase A: methanol/water/formic acid (74/25/1 v/v/v); mobile phase B: methanol/formic acid (99/1 v/v), both containing 5 mM ammonium formate. A linear gradient was programmed—0.0 min: 80% B; 3 min: 90% B; 6 min: 90% B; 15 min: 99% B;18 min: 99% B; 20 min: 80% B. The flow rate was 0.3 mL/min. The column was kept at 30 °C.

Quantification was carried out using the extracted ion chromatogram of each compound, in a 50 mDa windows. The linear dynamic range was determined by injecting standard mixtures. Positive identification of compounds was based on the accurate mass measurement with an error <5 ppm and its LC retention time, compared to that of a standard (±2%). Mass spectra were analyzed by MassLynx™ 4.1 Software and data were compared performing unpaired two-tailed Student’s t test, p-value < 0.05 using SigmaStat v.3.5 build.3.5.0.74 (Systat Software Inc., California, USA).

### Western blot analysis

Prior to SDS page analysis, CSF total protein content was determined using Pierce BCA protein assay Kit. Sera were randomly sub-pooled into two groups for iNPH and two groups for AD, each one constituted of 3 different samples. The protein content of the pooled CSF samples ranged from 0.5822 μg/μl to 1.204 μg/μl. 80 µg of CSF total proteins from each different sub-pool were separated in duplicate by sodium dodecyl sulphate - polyacrylamide gel electrophoresis (SDS-PAGE), transferred and blocked onto a polyvinylidene fluoride (PVDF) membrane (300 mA, 180 min) utilizing a Transblot Cell from GE Healthcare (Uppsala, Sweden). The membrane was blocked overnight in tris-buffered saline (TBS) (20 mM Tris, 137 mM NaCl, 0.1% Tween, pH 7.5) containing 5% bovine serum albumin (Sigma Aldrich, Saint Louis, MO, USA) and incubated with the following primary antibodies: anti-ASM 1:500 (Santa Cruz Biotechnology, sc-9817, Dallas, MO, USA), anti-nSMase 1:1000 (Abcam, ab-131330, Cambridge, UK) and anti- ASAH2 1:1000 (SigmaAldrich, PRS4743). Anti-goat (Santa Cruz Biotechnology, 1:5000) was used as secondary antibody for ASM, whereas anti-rabbit (GE Healthcare,1:10000) was used for nSMase and ASAH2. Proteins were visualized by chemiluminescence (ECL Prime kit, GE Healthcare). Band intensities were detected using an Image Quant LAS 4000 mini imager (GE Healthcare) and assessed with the Image Quant TL 8.1 analysis software (GE Healthcare). Data were normalized against the total amount of proteins stained by Sypro Ruby and Student’s t-test by comparing AD and iNPH. Differences were considered significant at p-value < 0.05.

## Electronic supplementary material

Supplementary Information

## Data Availability

The data generated during and/or analysed during the current study are included in this published article (and its Supplementary Information files). When needed, further information are available from the corresponding author on reasonable request.
