# Author Correction: Particular CSF sphingolipid patterns identify iNPH and AD patients

**DOI:** 10.1038/s41598-020-66184-6

**Published:** 2020-06-11

**Authors:** Enrica Torretta, Beatrice Arosio, Pietro Barbacini, Martina Casati, Daniele Capitanio, Roberta Mancuso, Daniela Mari, Matteo Cesari, Mario Clerici, Cecilia Gelfi

**Affiliations:** 1 Department of Biomedical Sciences for Health, University of Milan, Segrate, Milan, Italy; 2 Geriatric Unit, Department of Medical Sciences and Community Health, University of Milan, Milan, Italy; 3 Fondazione IRCCS Ca’ Granda – Ospedale Maggiore Policlinico, Milan, Italy; 4 Don C Gnocchi Foundation IRCCS, Milan, Italy; 5 Department of Physiopathology and Transplants, University of Milan, Milan, Italy; 6 Clinical Proteomics Unit, Scientific Institute for Research, Hospitalization and Health Care (IRCCS), Policlinico San Donato, San Donato Milanese, Milan, Italy

Correction to: *Scientific Reports* 10.1038/s41598-018-31756-0, published online 11 September 2018

In the original version of this Article, Mario Clerici was incorrectly affiliated with ‘Don C. Gnocchi Foundation-ONLUS, IRCCS, Milan, Italy’. The correct affiliations for this author are listed below.

Don C Gnocchi Foundation IRCCS, Milan, Italy

Department of Physiopathology and Transplants, University of Milan, Milan, Italy

This error has now been corrected in the PDF and HTML versions of the Article, and in the accompanying Supplementary Information file.

