# Supplementary material for: Particular CSF sphingolipid patterns identify iNPH and AD patients
Source: Sci Rep. 2018 Sep 11;8:13639. doi: 10.1038/s41598-018-31756-0 (PMC6133966; doi:10.1038/s41598-018-31756-0)
Supplement: Supplementary file 1 — Supplementary Information [file 41598_2018_31756_MOESM1_ESM.docx]

Supplementary Information

**Particular CSF sphingolipid patterns identify iNPH and AD patients.**

**Enrica Torretta^1^, Beatrice Arosio^2,3^, Pietro Barbacini ^1^, Martina Casati ^2^, Daniele Capitanio^1^, Roberta Mancuso^3^, Daniela Mari^2^, Matteo Cesari^2,3^, Mario Clerici^4,5^, Cecilia Gelfi^1,6*^**

1 Department of Biomedical Sciences for Health, University of Milan, Segrate (Milan), Italy.

2 Geriatric Unit, Department of Medical Sciences and Community Health, University of Milan, Milan, Italy

3 Fondazione IRCCS Ca’ Granda – Ospedale Maggiore Policlinico, Milan, Italy

4 Don C Gnocchi Foundation IRCCS.

5 Department of Physiopathology and Transplants, University of Milan, Milan, Italy.

6 Clinical Proteomics Unit, Scientific Institute for Research, Hospitalization and Health Care (IRCCS) Policlinico San Donato, San Donato Milanese (Milan), Italy.

*Corresponding Author:

Cecilia Gelfi, Professor

Department of Biomedical Sciences for Health, University of Milan, Via Fratelli Cervi 93, Segrate

(Milan), Italy.

Tel: +39 02 50330475.

Fax: +39 02 21717558.

e-mail: cecilia.gelfi@unimi.it

| **#sample** | **Diagnosis** | **Age (years)** | **Aβ (pg/mL)** | **Tau (pg/mL)** | **p-Tau (pg/mL)** | **MMSE** |
| --- | --- | --- | --- | --- | --- | --- |
| 1 | HC | 77 | 453 | 49 | 10 | - |
| 2 | HC | 73 | 581 | 54 | 7 | - |
| 3 | HC | 78 | 457 | 111 | 13 | - |
| 4 | HC | 77 | 853 | 85 | 14 | - |
| 5 | HC | 80 | 965 | 96 | 25 | - |
| 6 | HC | 84 | 1515 | 115 | 30 | - |
| 7 | HC | 74 | 1306 | 103 | 26 | - |
| 8 | HC | 70 | 1001 | 158 | 40 | - |
| 9 | HC | 68 | 1424 | 95 | 31 | - |
| 10 | HC | 74 | 1082 | 507 | 80 | - |
| 11 | iNPH | 83 | 1248 | 125 | 26 | 29 |
| 12 | iNPH | 86 | 790 | 157 | 28 | 28 |
| 13 | iNPH | 70 | 1010 | 130 | 15 | 23 |
| 14 | iNPH | 87 | 665 | 175 | 37 | 27 |
| 15 | iNPH | 82 | 610 | 75 | 15 | 29 |
| 16 | iNPH | 100 | 418 | 250 | 26 | 27 |
| 17 | iNPH | 86 | 661 | 676 | 73 | 21 |
| 18 | iNPH | 91 | 456 | 934 | 128 | 17 |
| 19 | iNPH | 87 | 859 | 284 | 42 | 24 |
| 20 | iNPH | 77 | 438 | 83 | 15 | 29 |
| 21 | AD | 70 | 307 | 609 | 124 | 25 |
| 22 | AD | 73 | 372 | 1420 | 132 | 25 |
| 23 | AD | 76 | 391 | 999 | 136 | 24 |
| 24 | AD | 71 | 493 | 1316 | 87 | 27 |
| 25 | AD | 76 | 393 | 1266 | 70 | 13 |
| 26 | AD | 76 | 462 | 745 | 64 | 21 |
| 27 | AD | 75 | 544 | 803 | 100 | 16 |
| 28 | AD | 82 | 354 | 567 | 81 | 26 |
| 29 | AD | 82 | 509 | 512 | 75 | 25 |
| 30 | AD | 73 | 456 | 934 | 128 | 18 |
| 31 | AD | 80 | 420 | 526 | 66 | 24 |
| 32 | AD | 78 | 226 | 423 | 80 | 21,3 |
| 33 | AD | 75 | 590 | 721 | 65 | 16 |
| 34 | AD | 80 | 322 | 208 | 28 | 19 |
| 35 | AD | 81 | 563 | 826 | 109 | 22 |
| 36 | AD | 76 | 546 | 491 | 101 | 27 |

**Table 1S** *Detailed participants’ characteristics.*


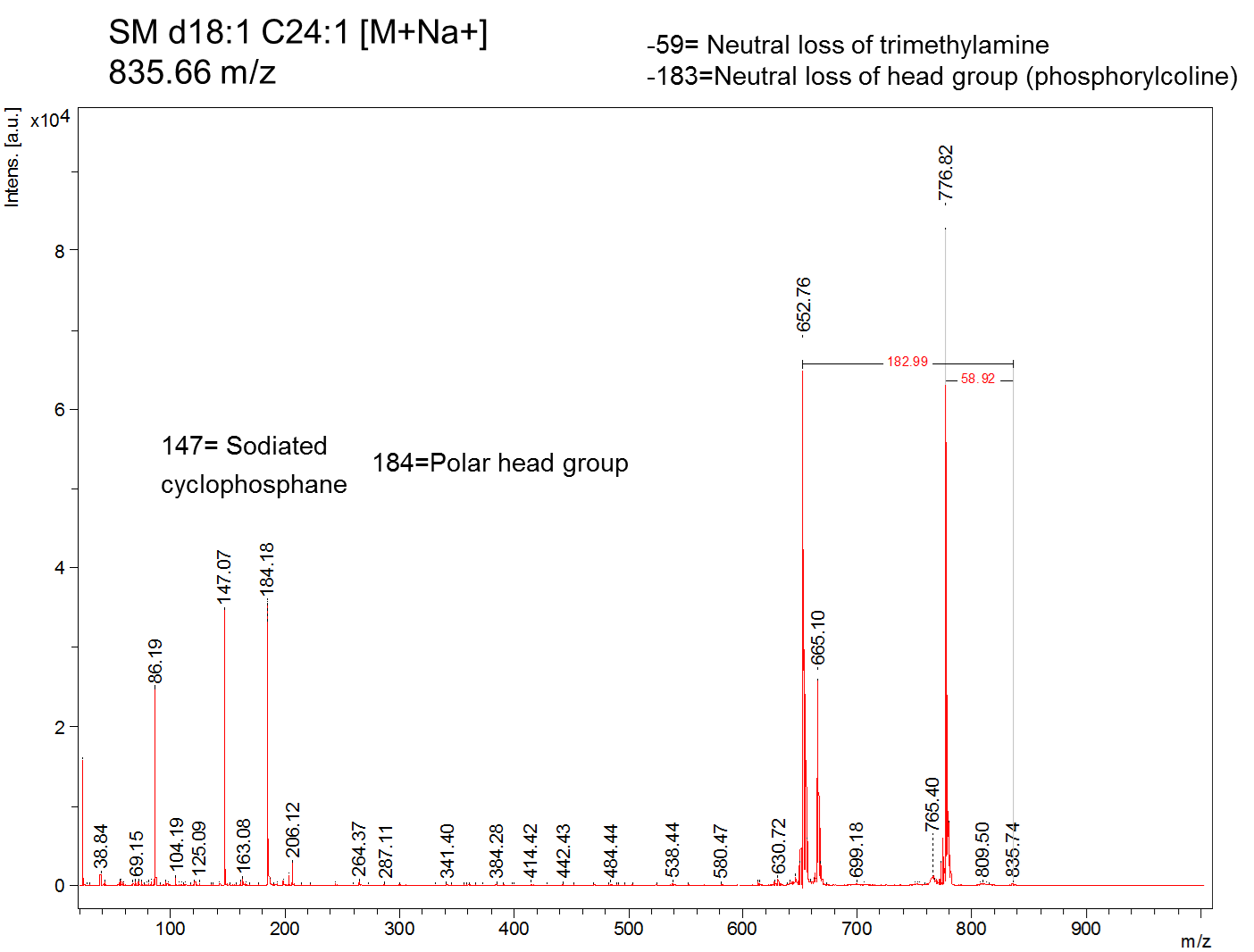


**Figure 1S** *MALDI MS/MS spectrum of the ion 835.68 m/z, identified as sphingomyelin d18:1 C24:1 [M+Na]^+^*


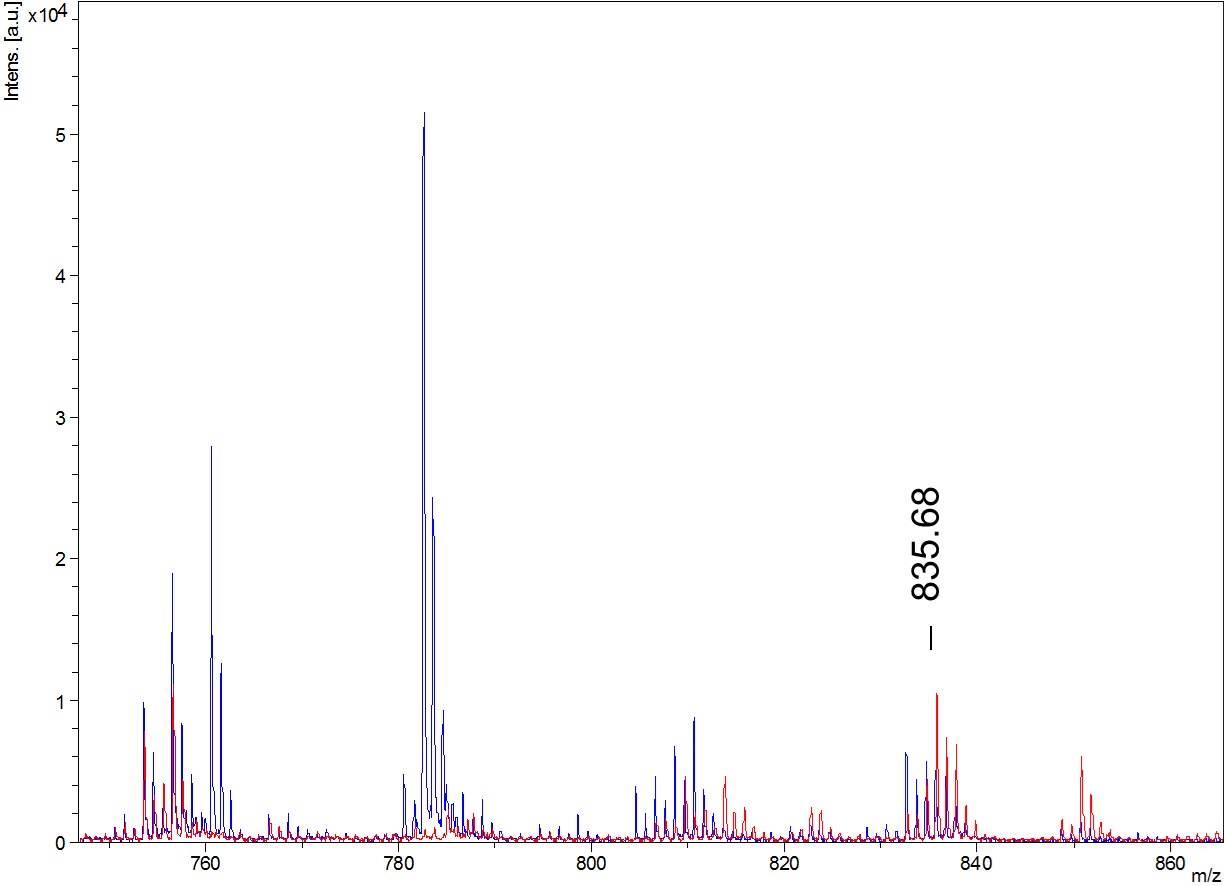


**Figure 2S** *MALDI-TOF-MS analysis of lipids extracted from serum organic phase, before the alkaline treatment (blue spectra) and after the reaction (red spectra). The two organic fractions (1μL) were loaded on GroundSteel plate (Bruker Daltonics) with DHB matrix at 20 mg/mL in 70% acetonitrile*

**Figure 3S** *MALDI MS/MS spectrum of the ion 808.61 m/z, identified as PC 36:2 [M+Na]^+^ through the NIST MS Search v 2.2 software*.

B


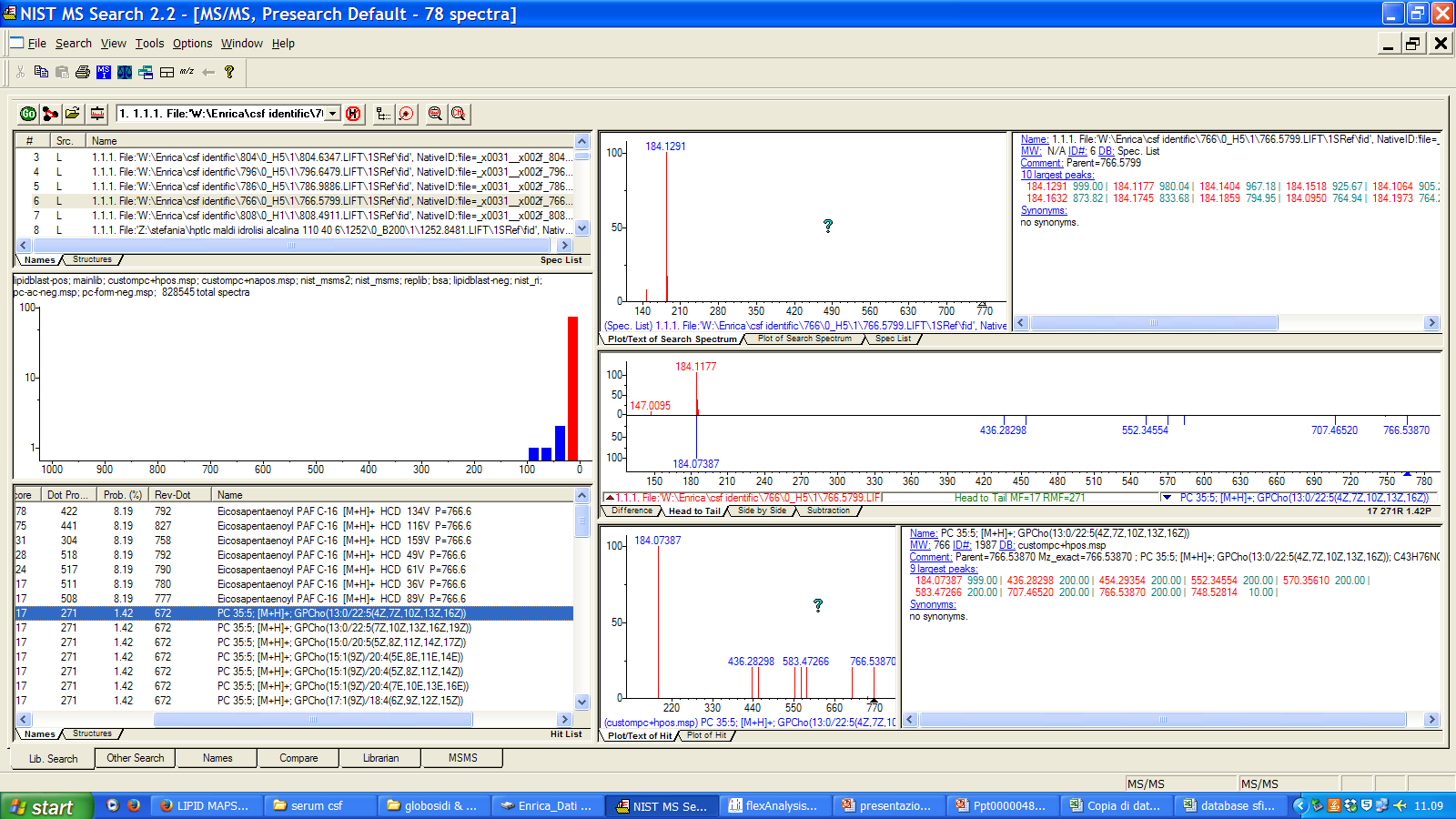


A

**Figure 4S** MALDI MS/MS spectra of the ion at 766.576 m/z (A), and of the ion at 796.591 m/z (B). In both cases, the NIST MS Search v 2.2 software provided multiple structures: PC(O-16:0) [M+H^+^]; PC 35:5 [M+H^+^] and PC 33:2[M+Na^+^] for the peak at 766.576 m/z and PC 36:8 [M+Na^+^]; PC 37:4 [M+H^+^] and PC 35:1[M+Na^+^] for the peak at 796.591 m/z.

**
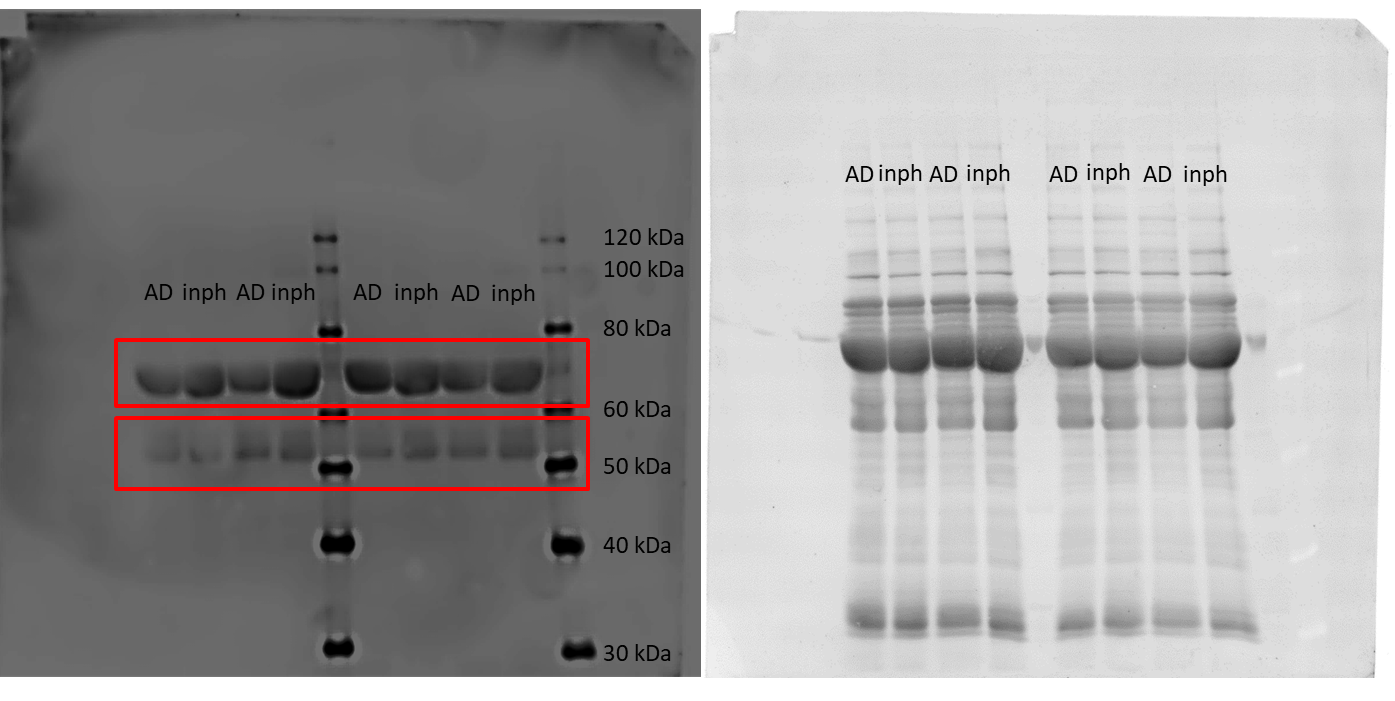
Figure 5S** (A) Full lenght blot of acid sphingomyelinase (ASM) (up) and neutral sphingomyelinase (nSMase) (down); red rectangles highlight bands that are shown in manuscript figures. (B) Data were normalized against the total amount of proteins stained by Sypro Ruby.

A

B
